# Dysregulation of heterochromatin caused by genomic structural variants may be central to autism spectrum disorder

**DOI:** 10.3389/fnmol.2025.1553575

**Published:** 2025-06-19

**Authors:** Michael R. Garvin, David Kainer

**Affiliations:** ^1^Williwaw Biosciences, LLC, Clarkston, MI, United States; ^2^Department of Chemistry and Chemical Biology, University of New Mexico, Albuquerque, NM, United States; ^3^Oak Ridge National Laboratory, Oak Ridge, TN, United States; ^4^Centre of Excellence for Plant Success in Nature and Agriculture, University of Queensland, Brisbane, QLD, Australia

**Keywords:** autism, structural variation, non-Mendelian inheritance, heterochromatin, multi-omics, chromatin, RITS, SATB1

## Abstract

**Introduction:**

Autism spectrum disorder (ASD) is a highly heritable and heterogeneous neuropsychiatric condition whose cause is still unknown. A common function of proteins encoded by reported risk-genes for ASD is chromatin modification, but how this biological process relates to neurodevelopment and autism is unknown. We recently reported frequent genomic variants displaying Non-Mendelian inheritance (NMI) patterns in family trios in two cohorts of individuals with autism. These loci represent putative structural variants (SV) and the genes that carry them participate in neurodevelopment, glutamate signaling, and chromatin modification, confirming previous reports and providing greater detail for involvement of these processes in ASD. The majority of these loci were found in non-coding regions of the genome and were enriched for expression quantitative trait loci suggesting that gene dysregulation results from these genomic disruptions rather than alteration of proteins.

**Methods:**

Here, we intersected these putative ASD-associated SVs from our earlier work with diverse genome-wide gene regulatory and epigenetic multi-omic layers to identify statistically significant enrichments to understand how they may function to produce autism.

**Results:**

We find that these loci are enriched in dense heterochromatin and in transcription factor binding sites for SATB1, SRSF9, and NUP98-HOXA9. A model based on our results indicates that the core of ASD may reside in the dysregulation of a process analogous to RNA-induced Initiation of Transcriptional gene silencing that is meant to maintain heterochromatin. This produces SVs in the genes within these chromosomal regions, which also happen to be enriched for those involved in brain development and immune response.

**Discussion:**

This study mechanistically links previously reported ASD-risk genes involved in chromatin remodeling with neurodevelopment and may explain the role of *de novo* mutations in ASD. Our results suggest that a large portion of the heritable component of autism is the result of changes in genes that control critical epigenetic processes.

## 1 Introduction

As we and others have reported, autism spectrum disorder (ASD) is highly heritable (50–80% is attributed to inherited genetic variants), yet no common or widespread gene or mutation has been found to be associated with autism in the broad sense, likely due in part to its complex heterogeneity (Gaugler et al., 2014; Bai et al., 2019; Ruzzo et al., 2019; Kainer et al., 2023). However, rarer variants have been identified in individuals or subgroups of those with autism, which are often reported as risk genes and the biological functions of their encoded proteins have provided insight into the condition as many participate in brain development (Nakanishi et al., 2019; Viggiano et al., 2024). One of the most commonly reported functions of the proteins encoded by these risk genes is the regulation of chromatin and they typically encode for subunits of the SWI/SNF (SWItch/Sucrose Non-Fermentable), NuRD (Nucleosome Remodeling Deacetylase), or ISWI (Imitation SWItch) complexes that open DNA for transcription by manipulating DNA-histone interactions.

A handful of genes participate in the opposing process of repressing DNA via methylation of lysine residues of histone 3 at position 9 (e.g., the H3K9 methyltransferase *EHMT1*, mutated in those with Kleefstra Syndrome). Despite their obvious importance in autism, there is currently no clear understanding or hypothesis of how genes involved in chromatin modification link to autism beyond generally affecting the expression of genes or epigenetic modifications to the DNA or histones (Neale et al., 2012; O’Roak et al., 2012a; O’Roak et al., 2014; Bernier et al., 2014; De Rubeis et al., 2014; Iossifov et al., 2014; Sun et al., 2016).

We recently demonstrated that a key source of genetic changes underlying ASD resides in genomic structural variants (SV). By focusing on SVs that were found at much higher frequency in people with ASD, we identified a regulatory element for the gene *ACMSD* that was statistically significantly associated with non-verbal forms of autism (Kainer et al., 2023). This provided important biological insights into ASD because the ACMSD enzyme functions to convert a toxic intermediate in the tryptophan salvage pathway (quinolinic acid) to a neuroprotective compound (picolinic acid). While this pathway has been previously linked to several neuropsychiatric conditions including ASD, that specific enzyme had not. Similarly, for another of these frequent SVs, we predicted and then confirmed abnormal splicing of the glutamate receptor subunit *GRIK2* in a subset of individuals with autism. This gene had also been linked to neuropsychiatric disorders including obsessive compulsive disorder and autism in those of Korean ancestry (Kim et al., 2007; Sampaio et al., 2011), and our work provided the molecular mechanism and specific genetic location as it relates to ASD. Using our entire matrix of genotyped SVs, we were able to identify subtypes of autism by clustering ASD cases according to their genomic SV profiles.

As just noted, in our previous work, intersecting SVs with coding genes provided better insight into their function in the context of ASD. Therefore, we reasoned that these SVs may also be the key to unlocking a clearer understanding of the role of chromatin modifying genes in the disorder by intersecting their genomic location with reported positions of eQTLs, heterochromatin, DNA and histone methylation, and transcription factor binding sites. We used diverse genome-level data sets in conjunction with a multi-omic approach to understand the functional genomics of the entire set of SVs we reported previously. Our results provide a potential explanation for the link between chromatin remodeling and brain development in autism.

We find that ASD-SVs are significantly enriched in constitutive heterochromatin and in binding sites for three transcription factors (SATB1, SRSF9, and NUP98-HOXA9) that regulate the formation of heterochromatin itself. This mechanistically links the SWI/SNF, NuRD, and ISWI complexes with what is analogous to the RNA-induced Initiation of Transcriptional gene Silencing (RITS) complex in yeast or RNA-Induced transcriptional Silencing Complex (RISC) in higher taxa. Previous work has determined that these multi-subunit complexes are meant to precisely balance suppression and expression of genomic regions that are rich in repetitive elements and transposons, which are also critical for human embryonic development (Yu et al., 2022; Wilkinson et al., 2023), but can be mutagenic if not tightly regulated. Interestingly, we find and report here for the first time that heterochromatin regions of the human genome are enriched for developmental genes. Our model indicates that an imbalance of this system therefore produces further SVs in genes critical to brain development. It may also explain the observation of *de novo* mutations in many cases of ASD, because loss of protective heterochromatin would result in a higher mutation rate.

## 2 Materials and methods

### 2.1 Study populations

The genomic data we used here and reported on previously (Kainer et al., 2023) were provided by the NIH database of Genotypes and Phenotypes (dbGAP) from two different studies. The first consisted of 1,177 individuals that represent 381 families, produced by researchers at the University of Miami (dbGAP accession phs000436.v1.p1, referred to as the MIAMI dataset) (Ma et al., 2009). The second study was produced by the Autism Genomic Project Consortium (dbGAP accession phs000267.v5.p2, referred to as the AGPC dataset), consisting of 4,168 individuals from 1,385 families (Anney et al., 2010). Both studies used the Illumina 1Mv1 SNP array, resulting in 1,048,847 nuclear SNP calls for MIAMI and 1,072,657 for AGPC. There were no overlapping families in the two studies.

### 2.2 ASD-enriched SVs

Numerous algorithms have been developed to use short-read sequencing to detect SVs but vary widely in their ability to detect certain types and are heavily influenced by variation surrounding the SV site (Cameron et al., 2019). Newer long read sequencing methods have identified twice as many SVs, indicating that critical genomic changes are still being overlooked in the search for the genetic causes of ASD when relying on short read callers (Liao et al., 2023). We previously reported an unconventional method to detect SVs that relies on non-Mendelian Inheritance (NMI) patterns generated from SNP genotyping arrays in family trios. It can be applied to large numbers of individuals and can capture SVs that short-read callers miss (Kainer et al., 2023). Furthermore, the fact that these loci appear to segregate in a non-Mendelian fashion means they are excluded from most analyses, yet we showed that they are likely true variants ranging from small to large effect (Kainer et al., 2023). Briefly, when there is genetic variation under the probe used for SNP genotyping arrays, they can cause the locus to violate Mendelian laws of inheritance because one of the alleles does not generate a proper signal. The assumption has been that the NMI signal is the result of technical errors of the genotyping platform, when it is actually caused by heritable genetic change. We and others have reported that many of these are genomic structural variants such as deletions and copy number variation that may be associated with or causative of a phenotype of interest, but they are currently being excluded from genomic studies because they are filtered during the quality control step (Conrad et al., 2006; Kainer et al., 2023).

**FIGURE 1:**
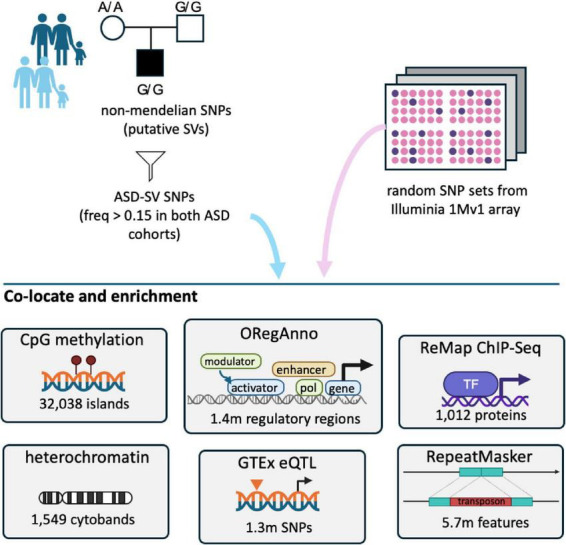
Overview of the analyses. ASD-SVs tagged with Non-Mendelian inherited (NMI) SNPs and found in greater than 15% of both the Miami and AGPC cohorts were extracted from the full database. These 2,468 SNPs, and the genes they potentially affect, were assessed for co-location and enrichment with a variety of genomic feature sets available on the UCSC table browser. For contrast, we randomly sampled the same number of SNPs 100 times from the remaining ∼900,000 SNPs on the Illumina array used for those studies, and performed the same analyses.

We produced a set of potential ASD-SVs that are most likely to represent the core of autism by using the NMI loci in the MIAMI population as a discovery set, and the AGPC population as a validation set. Our potential ASD-SVs were those SNPs that generated NMI signals in both populations (the overlap was roughly 90%, and the Pearson’s correlation coefficient between the SV frequency spectra of MIAMI and AGPC was 0.75). Most of these NMI SNPs were rare in the two populations. To produce a high confidence set of ASD-associated SVs, we removed any loci that were known SVs from the most recently reported non-ASD populations at a MAF > 0.02, we included only those SNPs that appeared in the two independent cohorts of children with ASD and we used only those NMI SNPs that were found in 15% or more of individuals in both studies (Kainer et al., 2023) leaving 2,468 that identified the potential “core” genomic structural variation of ASD (henceforth referred to as ASD-SVs, Figure 1, Supplementary Table S1). As we noted in our previous work, rarer NMI sites are more likely to be due to error than common NMI sites, so we removed all SVs with frequency less than 2% in the discovery population (MIAMI). We chose 2% because this is the estimated frequency of ASD in the human population but also an extremely conservative filter given that the technical error rate for the Illumina array used in this study was estimated to be less than 0.05%. The 2% NMI rate corresponds to seven individuals from the 380 families in the smaller study we used. Given the technical error rate of 0.05% the binomial probability of having an SNP assay fail at the same locus seven times in 380 trials is 1.4 × 10^–9^, where *p* = 0.05, *n* = 380, and *k* = 7. It should be noted that the QC of the Illumina bead arrays releases assays that display the technical error rate of 0.05% or less, i.e., it does not account for error rate due to the samples being analyzed. Therefore, by definition, the error rate of 2% is conservative given that it is 40 times higher than technical background error (Kainer et al., 2023).

### 2.3 Overlap of ASD-SVs with genomic features

Our genome-wide genomic feature enrichment analysis was based on the roughly 1 million sites queried by the Illumina 1Mv1 array from the original studies. We generated a *bed* file of intervals flanking ± 1 kb from each of the 2,468 NMI SNPs and the 100 randomly generated sets of the same size. We chose 1 kb to ensure that we captured variation in the regions surrounding genomic structural variants as it has been shown that mutation rates are higher in these regions as a result of repair after the event and therefore the causal mutation, as with standard GWAS, may be linked to the NMI locus (Massouras et al., 2012; Carvalho and Lupski, 2016). This is also consistent with the fact that the mean size of transcription factor binding sites listed in the ORegAnno data available on the UCSC genome browser is 294 basepairs. A 2 kb window will therefore capture nearby genomic features and is conservative.

The majority of the ASD-SVs we detected and reported previously (Kainer et al., 2023) were in non-coding space, which suggested that they affect regulatory or epigenetic mechanisms rather than protein function. Therefore, we intersected these intervals with the eQTL, heterochromatin (listed as gpos100, gpos75, gpos50, and gpos25), RepeatMask, CpG, ORegAnno, and ChIP-Seq data from RepMap Atlas (“a large-scale integrative analysis of all Public ChIP-seq data for transcriptional regulators from GEO, ArrayExpress, and ENCODE”) (Hammal et al., 2022) available from the UCSC Table browser using the Galaxy platform (Galaxy Community, 2022) because they represent different aspects of these non-coding processes. The Galaxy platform allows for the direct movement of data extracted from UCSC Table browser to Galaxy in the preferred format for any given tool and accounts for genome-versions. For the eQTL section, rather than the 2 kb overlap window, we queried for an exact match of the SNP locus. For the tissue-specific analysis, we binned tissues by organ (e.g., brain) where possible.

As a control, we did the same analysis for 100 sets of 2,468 SNPs randomly sampled (without replacement) from the loci listed on the Illumina 1Mv1 array used to generate the original MIAMI and AGPC datasets analyzed in our original report (Wang et al., 2009; Anney et al., 2010). Null distributions of genomic feature overlaps were generated from the 100 random SNP sets and compared to the ASD-specific set using a chi-square test and Bonferroni correction. Counts of any feature that were five or less at a site across a dataset were removed due to the inaccuracy of the chi-square test for these low values. For the analysis of the histone marks, we used a false discovery rate (FDR) < 0.05 for determining significance, calculated using a Benjamini-Hochberg test in base *R*.

### 2.4 Functional enrichment of genes regulated by TFs

We were interested in the function of the genes that are regulated by the transcription factors whose binding sites are disrupted by ASD-SVs more than expected by chance. The genes regulated by these transcription factors may be causal of autism because the ASD-SV that overlap their regulatory region from ReMap are frequent (*f* > 0.15) in both ASD cohorts. In order to ensure we captured the biological significance of these genes, we expanded the list to include ASD-SVs that were present in greater than 5% of both ASD cohorts and overlapped the three transcription factor binding sites found to be significantly enriched in our Chip-seq ReMap analysis (rather than just including common ASD-SVs that were greater than 15%). Many of these ASD-SV-tagging SNPs (*N* = 1,041, Supplementary Table S2a) are not in genic space and therefore we used several methods to assign them to a gene. First, we assigned to genes using the SNPnexus portal for Entrez (Dayem Ullah et al., 2018), unassigned genes were then queried for an exact rsID match in GTEx, and finally remaining unassigned genes intersected with ORegAnno 3.0 (Lesurf et al., 2016; Supplementary Table S2b). The protein coding genes were submitted to Gene Ontology^1^ for biological process (BP) term enrichment with a significance threshold FDR < 0.05. We also used a Gene Ontology BP enrichment for the genes found in the densest heterochromatin (gpos100). In this case, genes were assigned based on a bed file intersect of the gpos100 regions and gene locations from the UCSC Genome Browser. A gene was called “within” gpos100 if any portion of the gene was found within a gpos100 region.

### 2.5 Test for SATB1, SRSF9, and NUP98-HOXA9 DGE

Our results indicate that the genomic regions that harbor transcription factor binding sites for SATB1, SRSF9, and NUP98-HOXA9 are enriched for structural variants in individuals with autism. If this is correct, then one would expect the expression patterns for genes regulated by these transcription factors to differ compared to individuals without autism. In order to test this, we reanalyzed RNA-seq data from a previous study (Velmeshev et al., 2019). We examined the set of differentially expressed genes from post mortem brain tissue from subjects with autism and matched controls to determine if they were enriched for genes controlled by these transcription factors (Velmeshev et al., 2019). Reads were downloaded to CLC Genomics Workbench from the SRA database for project PRJNA434002 and mapped to human reference hg38 using a fraction score and similarity score of 0.95. Differential gene expression analysis was performed using counts normalized to library size. We included only genes that had a maximum group mean greater than 1 and excluded those on the X and Y chromosomes because they were not included in our original analysis using NMI. The analysis was performed separately on RNA taken from prefrontal cortex and anterior cingulate cortex and significant differences were those comparisons with an FDR < 0.05.

To determine if genes regulated by SATB1, SRSF9, and NUP98-HOXA9 were enriched in the differentially expressed genes overall, we calculated the expected number by dividing the total number of differentially expressed genes by the total number of genes tested. This frequency was then multiplied by the number of genes regulated by SATB1, SRSF9, and NUP98-HOXA9 (Supplementary Table S10a) that were expressed in each of the tissues with a maximum group mean greater than 1. The expectation was that the percentage of SATB1, SRSF9, and NUP98-HOXA9 controlled genes that were differentially expressed would be the same as the overall number of genes that were differentially expressed genome-wide. If, on the other hand, the binding sites for these transcription factors are altered by structural variants in those with autism, then the percentage of the SATB1, SRSF9, and NUP98-HOXA9 controlled genes that are differentially regulated should be greater than the percentage of all genes. We used a chi-square test for significance based on the observed differentially expressed genes linked to these transcription factors and the expected number based on the overall frequency of differentially expressed genes.

## 3 Results

### 3.1 Structural variant dataset

For this study, we used a set of SNP-array loci that tag putative structural variants at high frequency in ASD cohorts (Kainer et al., 2023). Briefly, we extracted all SNP loci that displayed patterns of non-Mendelian inheritance (NMI) in family trios from two studies available on the NIH dbGAP (Ma et al., 2009; Anney et al., 2010). The 48,009 NMI SNP loci that appeared in both cohorts were stringently filtered to a subset, referred to ASD-SVs, that were not already flagged as SVs in the broader population, and were found at a frequency of 0.15 or greater in the two ASD cohorts. The 2,468 high frequency potentially SV-tagging SNPs were assigned to 1,116 genes that were shown to be heavily enriched for brain development and chromatin regulation functions, and could be used to identify genetically distinct clusters of ASD cases (Kainer et al., 2023). These 2,468 loci therefore represent likely SVs that are enriched in the ASD population and therefore their association with genomic features available from numerous publicly available resources can further elucidate their biological role(s) in ASD.

### 3.2 Heterochromatin

The heterochromatin feature on the UCSC Table browser identifies seven regions: centromeric (acen) no heterochromatin (gneg), variable-length (gvar), and four levels of Giesma staining ranging from low to high (gpos25, gpos50, gpos75, gpos100). The SNPs that tagged ASD-SV were significantly enriched for the greatest staining intensity, i.e., the highest level of heterochromatin (gpos100, *p* < 1.4 × 10^–9^) and centromeric (acen, *p* < 8.3 × 10^–3^) compared to the null sets of SNPs which, in contrast, were significantly lower in euchromatin (gneg, *p* < 1.4 × 10^–3^) (Supplementary Table S3a).

**FIGURE 2:**
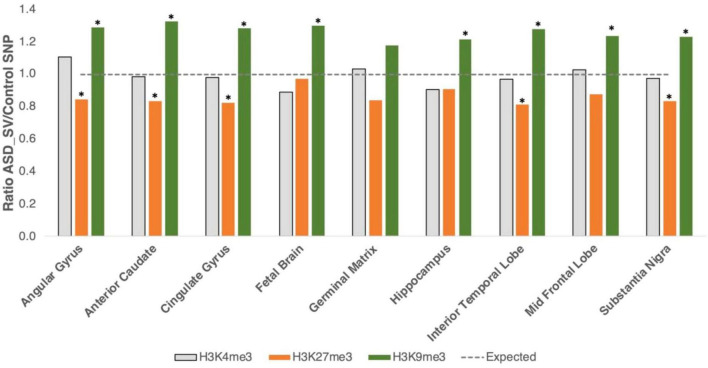
Intersection with specific histone marks. We generated bed files that represented the highest peaks for H3K4me3, H3K9me3, and H3K27me3 marks in human brain tissue from the ENCODE project. H3K4me3 marks euchromatin (gray bars), H3K27me3 is a marker for facultative heterochromatin (orange bars), and H3K9me3 for constitutive heterochromatin (green bars). Plot shows the ratio of ASD-SV tagging SNPs found in each layer divided by the mean of the 100 sets of control SNPs. Asterisks identify significant results (FDR < 0.05). Broken line is the expected ratio of 1.

To delve further into the role of heterochromatin as it relates to our ASD-SVs, we intersected their location with three different histone marks. Euchromatin is typically associated with tri-methylation at lysine residue 4 of the histone 3 protein (H3K4me3), facultative heterochromatin is associated with H3K27me3, and constitutive heterochromatin with H3K9me3. Data from eight brain regions and the whole fetal brain were available for analysis. In support of the broader analysis using Giemsa stain, H4K4me3 showed no significant differences between the ASD-SV tagging SNPs and the random set of controls (Supplementary Table S3b; Figure 2). However, there was significant enrichment of H3K9me3 marks in the ASD-SV subset in eight of the nine samples and a significant decrease in H3K27me3 marks. This indicates an association with constitutive, but not facultative, heterochromatin.

### 3.3 Repeat mask

**FIGURE 3:**
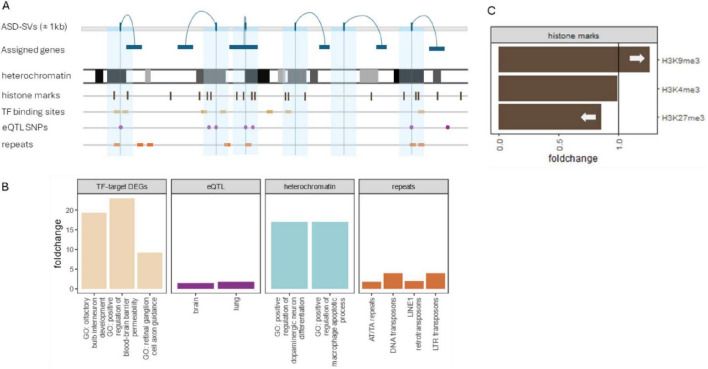
Summary of significant results. **(A)** SNPs showing patterns of non-Mendelian inheritance were assigned to genes and their locations were intersected with genomic tracks that included histone methylation, transcription factor binding sites, eQTLs from GTEx, and repeat mask elements from RepBase. **(B)** ASD-SVs were enriched in binding sites for three transcription factors (SATB1, SRSF9, and NUP98-HOXA9). A Gene Ontology (GO) enrichment of the genes affected by the ASD-SVs in those sites were enriched for several processes related to brain development (tan bars left). The ASD-SVs were also enriched in eQTLs that regulate transcripts in brain and lung (purple) and in several repetitive elements (orange far right). Although lung development is not typically associated with autism, there are reports of altered development of bronchii as a potential diagnostic (Stewart and Klar, 2013; Islam et al., 2024). Finally, the ASD-SVs were enriched in the densest heterochromatin based on Giemsa staining. A more detailed analysis of the heterochromatin using ChiP-Seq data from Encode revealed an enrichment of ASD-SVs in constitutive heterochromatin marked by H3K9me3 but a dearth in facultative heterochromatin marked by H3K27me3. Interestingly, we found a significant enrichment of genes involved in brain development and immune system processes in densest heterochromatin compared to euchromatin genome-wide (blue bars).

The repeat mask database lists 275 repetitive sequences taken from the RepBase update assembled by the Genetic Information Research Institute^2^ that are binned into 8 classes (Haddad, 2021). The ASD-SV SNPs were significantly enriched in 5 classes (DNA transposons, LINE elements, satellite DNA, simple repeats, and snRNAs, *p* < 8.1 × 10^–4^, Figure 3). There were no differences between the ASD-SV SNPs and the random SNPs for long terminal repeats, low complexity regions or SINE elements (Supplementary Table S4). When looking within each class, the significant LINE elements are type L1 and not L2, and the simple repeats are for AT or TA repeats (Supplementary Table S5). Type L1 LINE elements are younger than L2 and some are still active in the genome. They are responsible for retrotransposition of non-autonomous retrotransposons (e.g., *Alu*) as well as non-coding RNA and mRNA to produce pseudogenes (Beck et al., 2011).

### 3.4 Gene regulation

We tested for several regions associated with gene regulation and the ASD-SVs. CpG sites are often found in promoter regions of DNA and can result in gene regulation changes via differing methylation patterns as a result of mutations. We found no significant difference in the number of CpG sites between randomly chosen SNP loci and ASD-SV tagging SNPs (Supplementary Table S6). The Open Regulatory Annotation database (ORegAnno) is an open-source database for gene regulation curation. For the ASD-SV SNPs, there were fewer than expected overlaps of annotated ORegAnno sites overall (all transcription factor binding sites) as well as coding genes regulated by the transcription factors compared to randomly chosen SNPs (*p* < 8.8 × 10^–21^ and *p* < 6.0 × 10^–4^, respectively), suggesting that altered gene regulation is not the direct functional outcome of these mutations (Supplementary Table S6). However, in agreement with our previous report, they were enriched for eQTLs (*p* < 3.5 × 10^–18^). Here, we expanded this analysis to include tissue-specific tests and found that the ASD-SVs were significantly enriched for eQTLs in the brain (*p* < 6.4 × 10^–6^) and lung (*p* < 1.2 × 10^–3^). Finally, in general agreement with the ORegAnno results, the ChIP-Seq analysis with the 1,012 transcription factor binding sites in the ReMap Atlas indicates that the majority of these sites are either not different between the ASD-SV and control sets of SNPs, or are found significantly less frequently in the ASD-SV tagging SNPs. However, the binding sites of three specific transcription factors were significantly enriched in the ASD-SV tagging SNPs after Bonferonni correction: SATB1 (*p* < 2.7 × 10^–25^, *n* = 556), SRSF9 (*p* < 1.2 × 10^–8^, *n* = 119), and NUP98-HOXA9 (*p* < 1.1 × 10^–6^, *n* = 38) (Supplementary Table S8; Figure 3).

### 3.5 Gene ontology

Our analysis indicated ASD-SVs were found more frequently in dense heterochromatin. To determine if dense heterochromatin harbored genes of related functions, we performed a biological enrichment test on the coding-genes found in the gpos100 regions identified in the UCSC Table Browser (*N* = 1,194) and compared those to the same number of randomly sampled genes from euchromatin (nine sets after sampling without replacement, Supplementary Table S9). The genes found in dense heterochromatin were significantly enriched for specific functions related to immune response (e.g., positive regulation of macrophage apoptotic process) and brain development (e.g., positive regulation of dopaminergic neuron differentiation) both 17-fold enriched. Whereas seven of the nine euchromatin gene sets (*N* = 1,194 genes each, 10,476 total) were found to have no significant enrichments. Two of the nine were enriched for the broad and uninformative category of cellular process, 1.1-fold enriched.

The ASD-SVs were enriched in transcription factor binding sites for SATB1, SRSF9 and NUP98-HOXA9 (within the 2 kb window). This suggests that many genes controlled by these transcription factors may be dysregulated because of changes within or surrounding the binding site. There were 585 coding genes that were assigned to the ASD-SV SNPs within or near the binding sites for those transcription factors (Supplementary Table S2b). Sixty of the 585 were annotated with GO terms associated with chromatin, leaving 525 genes that represent non-chromatin processes related to ASD. GO enrichment of those 525 genes generated 36 significantly enriched top-level terms for Biological Processes (FDR < 0.05, Supplementary Table S10a). As with our previously published result (Kainer et al., 2023), dendritic spine development and glutamate receptor signaling featured prominently, plus developmental processes involving the retinal and olfactory systems. The process of *retinal ganglion cell axon guidance* was more than 9-fold enriched and *olfactory bulb development* more than 19-fold. Interestingly, the analysis identified 11 genes representing a 4-fold enrichment for female gonad development, which might potentially explain sex differences in diagnosed cases of ASD (Supplementary Table S10a).

### 3.6 Test for SATB1, SRSF9, and NUP98-HOXA9 DEG enrichment

If many of the 585 genes putatively targeted by these three transcription factors (SATB1, SRSF9, and NUP98-HOXA9) are more commonly dysregulated in autism because of disruption to the binding sites of the transcription factors, then we expect the 585 genes to show a greater rate of differential expression in autism cases compared to all expressed genes. In the anterior cingulate cortex tissue, a total of 13,900 genes were expressed, of which 671 were differentially expressed in those with autism, thus giving an overall expected DEG rate of 4.8%. In this tissue, 487 of the 585 target genes (Supplementary Table S10c) were expressed including 40 DEGs (8.2%). This rate of differential expression for the target genes was significantly greater than for all genes (1.7 fold increase, *p* < 6.4 × 10^–4^) (Figure 3).

On the other hand, in prefrontal cortex tissue, only 54 of the 13,768 expressed genes were differentially expressed in those with autism (FDR < 0.05) for an overall DEG rate of just 0.39%. While 481 of the 585 target genes were expressed in this tissue (Supplementary Table S10b), none were differentially expressed, which is close to the expected rate of 0.39%.

## 4 Discussion

Here we used a set of ASD-related SVs we had identified in our previous report (Kainer et al., 2023) and analyzed them in a genomic context, with the results indicating that the principally dysregulated process in ASD appears to be the maintenance of constitutive heterochromatin. We show significant over-representation of the ASD-SVs in heterochromatin and under-representation in euchromatin. The ASD-SVs are also more often found overlapping features known to be associated with heterochromatin such as ALR-alpha satellite DNA, transposons, small nuclear RNAs, and simple repeats. Although genes involved in chromatin remodeling have been reported in autism previously and some overlap with our ASD-SVs (Supplementary Table S11; Vorsanova et al., 2007; Sokpor et al., 2017; Gabriele et al., 2018), our results here suggest that the key process is specifically chromatin remodeling as it relates to the *regulation of heterochromatin* rather than gene regulation or epigenetics in the broader sense. This hypothesis is based on the fact that (1) known causative genes for autism subtypes are components of the multi-subunit complexes that carry this process, which was likely overlooked previously due to the confusing nomenclature of the system, (2) the SATB1 protein that targets one of our significantly enriched transcription factor binding sites is a component of this same system, and (3) the remaining two proteins are likely regulators of the RNA intermediate necessary to carry out this process. Below we provide a clearer and more holistic understanding of how this biological process relates to downstream neurodevelopment.

### 4.1 Heterochromatin formation

Heterochromatin is generated by epigenetic modifications of the histone proteins around which the DNA is wrapped, resulting in compaction and inaccessibility of the genome to transcription or replication. This is necessary because these regions of the DNA harbor repetitive sequences or transposable elements that could generate SVs and genomic disruptions through either active transposition or non-homologous recombination (Volpe et al., 2002; Grewal and Jia, 2007; Saksouk et al., 2015). However, these regions are not simply “junk” but also harbor genes that code for protein and must be accessible to transcriptional machinery at certain times. This likely drove an evolutionary response to repress parasitic genomic elements such as transposons while simultaneously allowing for the tightly controlled expression of genes in those same regions that are important for development and cellular differentiation (Becker et al., 2016; Allshire and Madhani, 2018).

Amazingly, even though only 1% of the genome encodes for proteins, nearly three-quarters of the genome is transcribed into RNA (Djebali et al., 2012; The Encode Project Consortium, 2012). Much of this material is used for the regulation of heterochromatin, which is typically characterized as facultative heterochromatin and constitutive heterochromatin. Facultative heterochromatin is associated with tri-methylation of histones at the lysine 27 residue (H3K27me3) whereas constitutive heterochromatin is associated with tri-methylation of histones at lysine residue 9 (H3K9me3) and is fully methylated throughout the cell cycle (Saksouk et al., 2015). H3K27 is regulated by the polycomb repressive complex (PRC) whereas H3K9me3 is maintained by a system that uses small interfering RNA (siRNA or RNAi) that result in compaction of DNA and inaccessibility by transcription machinery via complementary base pairing (Cam and Grewal, 2004; Grewal and Jia, 2007; Martienssen and Moazed, 2015; Bhattacharjee et al., 2019). The system was originally elucidated in yeast (*S. pombe*) and termed “RNA-induced initiation of transcriptional gene silencing” (RITS) but due to inconsistent gene nomenclature, it has been difficult to make direct comparisons in mammals. Indeed, more than 100 genes participate in the function of the PRC and the mammalian equivalent of RITS, many of which have multiple different names reported in the literature (Supplementary Table S11; Martienssen and Moazed, 2015). Two analogous systems in mammals are RNA-induced silencing complex (RISC) and piwi-interacting RNAs (piRNAs). Although the exact details are still being debated, it is clear that the formation of heterochromatin involving histone methylation and transcribed RNA intermediates is important for early stages of development in the mammalian embryo (Santenard et al., 2010; Burton et al., 2020; Fioriniello et al., 2020; Stamidis and Żylicz, 2023).

**FIGURE 4:**
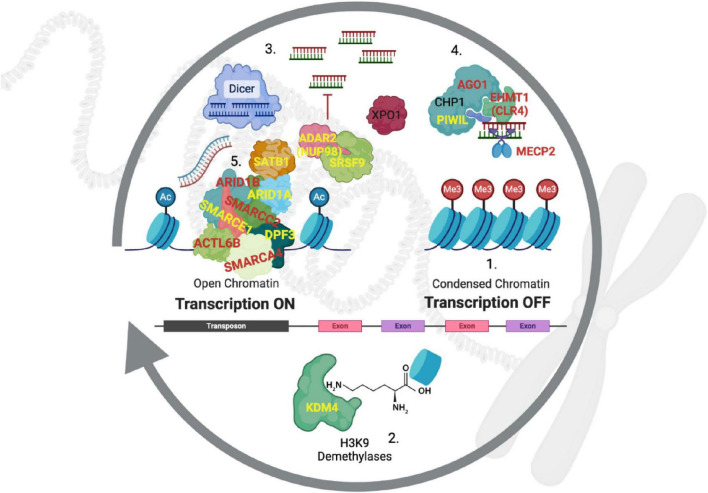
Heterochromatin regulation by RITS/RISC. Historically, gene nomenclature has been confusing and required translation from other species and systems (Supplementary Table S11). (1) Constitutive heterochromatin is maintained in a repressed state with the tri-methylation of the lysine-9 residue on histone 3 (H3K9me3). (2) Demethylases from the KDM4 family remove these repressive methyl groups to expose heterochromatin-rich genomic regions. (3) DICER cleaves double stranded RNA (both strands are transcribed) to generate short sequences that then bind to argonaut proteins (e.g., AGO1), which (4) direct the RITS complex back to the original genomic locations where bound H3K9 methyltransferases (EHMT1) re-establish suppression by writing H3K9me3 back to histones. (5) Occupancy of the DNA by chromatin-modifying complexes such as SWI/SNF counteract the re-suppression by the RITS complex to allow for transcription of genes in these regions. Genes in red are known to be causative of subtypes of autism, those in yellow are linked to high frequency ASD-SVs in this study and our previous report. PIWIL1 is also a component of RITS analogous to argonaut proteins (Gunawardane et al., 2007), but it is typically expressed in the germline to suppress transposons as well as genes (Meister, 2013).

Access to these regions begins with the removal of methylation at H3K9 residues by demethylases (i.e., erasers) that open the local genome to transcription. Reversion to a quiescent state is accomplished by converting the transcribed genomic regions to small RNA oligos by DICER, which are then used to target the RITS/RISC complex back to the initial location of transcription so that H3K9 methylases (i.e., writers) can re-methylate the histones (Figure 4). In the context of disease, it is important to note that if this process is not tightly controlled, heterochromatin spreads into neighboring genomic regions, causing their suppression. This is called position effect variegation (PEV), and was discovered as the causative process of the white-eye phenotype in *Drosophila* because a transposition in that mutant resulted in heterochromatin being placed next to the *white* gene, causing it to be silenced (Girton and Johansen, 2008).

### 4.2 Linking heterochromatin regulation to ASD

Notably, two subtypes of autism are caused by mutations in the proteins that carry out the formation of heterochromatin. Kleefstra Syndrome is the result of mutations in the H3K9me3 writer *EHMT1* (also called *CLR4* or *G9A*) (Frega et al., 2019) and Rett Syndrome is caused by mutations in the *MECP2* gene that codes for a DNA-methylase that modifies the DNA where it contacts H3K9 residues (Fuks et al., 2003; Thambirajah et al., 2012; Bian et al., 2019). Autism-like syndromes have also been reported in cases of *de novo* deletions of *AGO1* (numeral 4, Figure 4) (Sakaguchi et al., 2019; Niu et al., 2022). We, and others, have identified the H3K9me3 demethylases *KDM4B* and *KDM4C* as risk-genes for ASD (numeral 2, Figure 4, Supplementary Table S11; De Rubeis et al., 2014; Kainer et al., 2023). Here we add to this well-established model, three transcription factors (SATB1, SRSF9, and NUP98-HOXA9) whose binding sites are enriched for ASD-SVs and participate in the maintenance of heterochromatin, as well as enrichment of ASD-SVs in H3K9me3 regions of the genome, but not in H3K4me3 or H3K27me3.

Reports from more than 2 decades ago established that *SATB1* is associated with H3K9 methylation, heterochromatin, and brain-specific gene regulation (Cai et al., 2003). It has also been shown to recruit subunits from NuRD and ISWI complexes (Yasui et al., 2002). Later work established that it balances activation and repression states of heterochromatin by regulating acetylation versus methylation of H3K9 (Kohwi-Shigematsu et al., 2013). More recent work determined that it is necessary for *X-ist* mediated X inactivation during embryogenesis in which one X chromosome in each cell of a female is silenced by converting the entire chromosome to a state of constitutive heterochromatin (Wutz, 2011). Our analysis here indicates that the ASD-SVs are 1.5 times as likely to be associated with SATB1 binding sites than expected by chance. SATB1’s binding site is also the most common of the three significant binding sites predicted to be disrupted (*p* < 2.7 × 10^–25^, Supplementary Table S8). In support of our results here, haploinsufficiency of *SATB1* itself has been reported to cause an autism-like neurodevelopmental condition. Therefore, it stands to reason that disruption of DNA at an excessive number of SATB1 target motifs could drive a range of ASD-like conditions, as heterochromatin regulation would differ in these regions compared to non-ASD individuals (Bissell et al., 2022).

In our previous study, we predicted aberrant splicing of the *GRIK2* gene due the presence of an ASD-SV SNP near a known binding site for SRSF9, and subsequently confirmed the prediction using RNA-seq data from post-mortem brain tissue from individuals with autism (Velmeshev et al., 2019; Kainer et al., 2023). In addition to its role as a component of the spliceosome, SRSF9 regulates brain-specific editing of mRNA by the enzyme ADAR2 (also called NUP98, see third transcription factor below) that is necessary for normal neuronal development (Krestel and Meier, 2018; Shanmugam et al., 2018). This often occurs at *Alu* retrotransposons, which can affect the stability and splicing of the mRNA transcripts directly, or alter expression by affecting the formation of heterochromatin by destabilizing siRNAs (Savva et al., 2012; Savva et al., 2013; Figure 4). A hypothesis consistent with our results is that individuals with autism should carry SVs in binding sites for SRSF9 and therefore dysregulation of SRSF9-ADAR2-directed RNA-editing, which is supported by reports of decreased editing in post-mortem brain tissue in those with ASD compared to controls (Tran et al., 2019).

A common non-homologous recombination event between the *NUP98* gene on chromosome 11 (also called *ADAR2*) and *HOXA9* on chromosome 7 can result in a fusion protein that results in the expression of the homeobox locus genes downstream of *HOXA9*, in addition to many other genes. Although there are no reports of this fusion event in ASD, several lines of evidence support the role of this fusion protein in autism and support our model here. Firstly, as noted above, the NUP98 protein (also called ADAR2) physically associates as a protein complex with SRSF9 and mRNA to regulate editing of neuronal-specific genes (Shanmugam et al., 2018). Secondly, the fusion protein along with the protein XPO1 targets heterochromatin (Oka et al., 2016), and thirdly, microdeletions of *XPO1* cause ASD-like syndromes (Liu et al., 2011). In addition, one of the ASD-SV SNPs (rs9695393) that overlaps a NUP98-HOXA9 binding site is adjacent to the microRNA mir-873, which is known to reduce the expression of ASD risk genes *ARID1B* (a component of SWI/SNF), *SHANK3* and *NRXN2* (Lu et al., 2020). In addition, our previous report that identified our ASD-SVs used here reported two other frequent ASD-SVs in this genomic region that did not make our list here because it was not found at 15% or more in both data sets (rs3904396 found at frequencies of 0.14 and 0.22 and rs1928661 found at 0.09 and 0.12). These ASD-SVs bracket several features that regulate mir-873.

Finally, we re-analyzed previously reported results from RNA-seq data from post mortem brain tissue in those with autism (Velmeshev et al., 2019) and find that a greater number of the genes that are regulated by these three transcription factors are differentially expressed in the anterior cingulate cortex than would be expected (Supplementary Table S10c). This is consistent with disruptions of the transcription factor binding sites in those with autism. An alternative explanation for this result would be that the transcription factors themselves are altered in some way, but there have been no reports of protein coding disruptions in those with autism nor did we identify any ASD-SVs that were frequent near these genes.

### 4.3 A mechanistic model of heterochromatin-driven ASD

A hypothesis consistent with our results is that dysregulation of constitutive heterochromatin is a core biological process of autism spectrum disorder, which directly affects coding-genes that reside in these locations. Our Gene Ontology analysis of genes residing in the densest heterochromatin regions of the human genome (gpos100) show significant enrichment for neurodevelopment whereas those in euchromatin show no significant biological enrichments (Supplementary Table S9). This heterochromatin analysis is based on Giemsa staining, which is a fairly course description of the state of chromatin and therefore we also tested for differences in overlap of our ASD-SVs using histone marks. Those results also support this hypothesis, given that we found an enrichment of ASD-SVs regions marked with dense H3K9 trimethylation (constitutive heterochromatin), but a reduction of them in regions of H3K27 trimethylation (facultative heterochromatin), and no difference in regions of H3K4 trimethylation (euchromatin). Genes located within constitutive heterochromatin are normally repressed due to the presence of transposons and repetitive DNA elements because they can cause genomic instability. Recent studies have shown that the proteins that regulate heterochromatin are some of the most rapidly evolving in any organism to counter the high mutation rate of the DNA within heterochromatin itself (Brand and Levine, 2021). Although RITS/RISC-like processes that regulate constitutive heterochromatin likely represent this rapid evolutionary response to counter deleterious mutations, it may have also become an efficient means to regulate gene expression and diversity for the genes that reside in these locations. In other words, transposons and repetitive elements in the genome necessitated the evolution of a system to tightly regulate when those genomic regions need to be expressed because there are coding genes within those locations too.

SVs in these regions would be predicted to disrupt this important regulatory process and the evolutionary rate of the genes located within due to a higher mutation rate. Seen in this manner, autism is the phenotypic expression of genetic variants generated within rapidly evolving regions of the genome that are likely directed at the evolution of larger brains in the Hominid lineage as noted previously (Polimanti and Gelernter, 2017). In support of this model, an ASD-SV tagged at rs1957862 in the *LRFN5* gene (Supplementary Table S2a) is found in nearly one-third of individuals in both the MIAMI and AGPC cohorts we analyzed previously (Kainer et al., 2023). This is an ASD susceptibility locus, is involved in synaptic development, and was shown to be differentially marked with H3K9me3 in male individuals with autism when maternally inherited (Lybaek et al., 2022).

Mechanistically, the proposed model that regulation of constitutive heterochromatin may underlie the molecular basis of autism clarifies previous reports of the role of chromatin modifiers in the disorder (O’Roak et al., 2012b; Iossifov et al., 2014; Ruzzo et al., 2019). Once the suppressed heterochromatin state has been relaxed by removal of the H3K9 trimethylation, these DNA regions are exposed to ATP-dependent chromatin remodeling complexes such as SWI/SNF, ISWI, and NuRD (Sokpor et al., 2017). Mutations in subunits important for the function of these complexes have been associated with, or are causal of some cases of autism (Neale et al., 2012; O’Roak et al., 2012b; Iossifov et al., 2014). We also found numerous ASD-SVs in these genes in our previous work and report them here (Supplementary Table S11). These ATP-dependent chromatin remodelers act to balance suppression and activation of heterochromatin because their depletion results in the rapid return to the quiescent state (Stanton et al., 2017). Therefore, any mutations that alter the levels or efficiency of these chromatin-modifying complexes will affect the expression of the genes in those locations as well as the state of heterochromatin in adjacent genomic regions, again emphasizing the importance of heterochromatin in the etiology of ASD.

**FIGURE 5:**
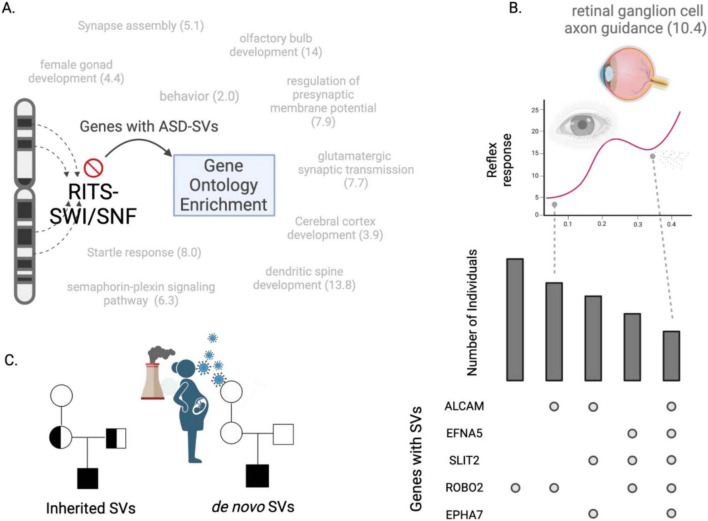
Heterochromatin-centric model for ASD. **(A)** Dysfunctional RITS and/or SWI/SNF processes cause structural variants to occur in genes within heterochromatin rich regions. A Gene Ontology (GO) analysis of the genes harboring SVs at high frequency in two cohorts reveal biological processes associated with ASD, such as glutamate signaling and startle response. Numbers indicate fold-enrichment of the ASD-SV genes for those GO biological processes compared to a random set of genes. **(B)** The genes that define these biological processes can be used to develop gene- and individual-focused diagnostic tests. The upset plot (lower right) shows frequency of individuals (y-axis) and the combination of genes with an SV that define the GO category “retinal ganglion axon guidance” (barplot here is for demonstration purposes, true barplot is in Supplementary Table S12). The pupil dilation test for autism may be more precise when accounting for the genetic contributions to the trait. For example, individuals with SVs in ALCAM and ROBO2 may have a lower reflex response than individuals with SVs in all five genes. **(C)** Our results suggest a dysfunctional RITS-SWI/SNF system underlies ASD by generating SVs that then segregate like any genomic variation from parents to children. This disrupted RITS-SWI/SNF pathway may also explain the role of *de novo* mutations in autism as they would be an indirect measure of heritability, i.e., they are the result of the inherited mutations in RITS-SWI/SNF that then produce new mutations. This could be exacerbated by the exposome during development.

In summary, we hypothesize that dysregulation of heterochromatin maintenance is a core mechanism leading to ASD. As a result, there is an increase in mutations and generation of structural variation in the genes within the heterochromatin-rich regions of the genome, resulting in an impact on the functions most commonly imparted by the genes in those regions. As shown above, dense heterochromatin is enriched for genes involved in brain development (Supplementary Table S9). Therefore, the functions of coding genes that are not chromatin-modifying, but do harbor ASD-SV and are regulated by SATB1, SRSF9, and NUP98-HOXA9, should provide key insights into the cause of different ASD phenotypes (Supplementary Table S10; Figure 5A).

In agreement with this, we found that such genes had a more than 10-fold significant enrichment for the GO BP term *retinal ganglion cell axon guidance*, which could explain the observation of reductions in the retinal nerve fiber layer in some individuals with autism (Emberti Gialloreti et al., 2014; Friedel et al., 2022). This may also underlie reported differences in pupil light reflexes as it is controlled by cells in this lineage (DiCriscio and Troiani, 2017; La Morgia et al., 2018; Lynch et al., 2022), which could be tested by comparing pupil responses of individuals carrying mutations in the genes we identified here (*ALCAM, EFNA5, SLIT2, EPHA7*, and *ROBO2*, Figure 5B; Supplementary Tables S12–S14) with individuals that do not. Likewise, the genes harboring ASD-SVs and linked to olfactory bulb development may explain differences in responses to odors in subsets of individuals with ASD and provide yet another diagnostic test (Xu et al., 2020).

Finally, these findings may help explain the role of environmental factors and *de novo* SVs in ASD. Several studies have identified a higher burden of *de novo* mutations in individuals with autism compared to typical developing individuals (Sebat et al., 2007; O’Roak et al., 2011; Neale et al., 2012; De Rubeis et al., 2014; Yuen et al., 2015). Although these findings are interesting, these mutations are not inherited and therefore would not contribute to estimates of heritability. However, if the core dysfunction in autism is the proper maintenance of heterochromatin, then its dysregulation could increase the frequency of *de novo* mutations due to the improper suppression of transposons and increased non-homologous recombination (Wiśniowiecka-Kowalnik and Nowakowska, 2019). *De novo* mutations therefore would be an indirect measure of heritability because they are the result of inherited dysfunctional genome (heterochromatin) maintenance. This could explain previous reports of no differences in transposon insertions in parents, probands, and unaffected siblings (Borges-Monroy et al., 2021) and support our model that ASD is the result of the epigenetic interaction among the heterochromatin-associated coding genes within each individual that were generated by the faulty RITS/RISC-BAF complex. Likewise, an epigenetic-driven mechanism of autism allows for contributions from the environment because stressors such as pollution or maternal infection during pregnancy can activate transposons and disrupt epigenetic processes that regulate heterochromatin (Saksouk et al., 2015; Figure 5C).

## 5 Limitations

Our results and conclusions are based on the current state of human genome research and therefore these may change over time as new information is added from efforts such as the Human Pan Genome Project.^3^ The two studies that formed the basis of our original analysis were mostly of European ancestry and therefore these results may not translate into individuals of non-European ancestry. Our results are based on knowing that the SV resides within a 50 base pair region of the SNP array probe, but the exact change at the DNA level is not known. Our mechanistic model of autism and heterochromatin is a hypothesis based on the set of loci identified in our previous study that will need to be further validated with other techniques and in regions outside the 1,000,000 queried here.

## 6 Conclusion

The results of our study indicate that the maintenance of constitutive heterochromatin is the core biological process that is disrupted in individuals with autism. The regions of the human genome that are the most heterochromatin-dense carry genes that are enriched for brain development. Our proposed model indicates that autism may be the result of dysregulation of the genes in these specific regions and the presentation of autism at the individual level would be the combination of the tens or hundreds of disrupted genes. This could partially explain the high heterogeneity and spectrum nature of the disorder.

## Data Availability

The data presented in this study were from public repositories available at the UCSC genome browser or are included in the supplementary tables. The original genomic data is available in the NIH Database of Genotypes and Phenotypes under accessions phs000436.v1.p1 and phs000267.v5.p2.

## Footnotes

1
www.geneontology.org/

2
www.girinst.org

3
www.humanpangenome.org
